# Detecting changes in facial temperature induced by a sudden auditory stimulus based on deep learning-assisted face tracking

**DOI:** 10.1038/s41598-019-41172-7

**Published:** 2019-03-18

**Authors:** Saurabh Sonkusare, David Ahmedt-Aristizabal, Matthew J. Aburn, Vinh Thai Nguyen, Tianji Pang, Sascha Frydman, Simon Denman, Clinton Fookes, Michael Breakspear, Christine C. Guo

**Affiliations:** 1 QIMR Berghofer Medical Research Institute, Brisbane, Australia; 2 School of Medicine, The University of Queensland, Brisbane, Australia; 3 Image and Video Research Laboratory, SAIVT, Queensland University of Technology, Brisbane, Australia; 4 School of Automation, Northwestern Polytechnical University, Xi’an, China; 5 The University of Newcastle, Newcastle, Australia

## Abstract

Thermal Imaging (Infrared-Imaging-IRI) is a promising new technique for psychophysiological research and application. Unlike traditional physiological measures (like skin conductance and heart rate), it is uniquely contact-free, substantially enhancing its ecological validity. Investigating facial regions and subsequent reliable signal extraction from IRI data is challenging due to head motion artefacts. Exploiting its potential thus depends on advances in analytical methods. Here, we developed a novel semi-automated thermal signal extraction method employing deep learning algorithms for facial landmark identification. We applied this method to physiological responses elicited by a sudden auditory stimulus, to determine if facial temperature changes induced by a stimulus of a loud sound can be detected. We compared thermal responses with psycho-physiological sensor-based tools of galvanic skin response (GSR) and electrocardiography (ECG). We found that the temperatures of selected facial regions, particularly the nose tip, significantly decreased after the auditory stimulus. Additionally, this response was quite rapid at around 4–5 seconds, starting less than 2 seconds following the GSR changes. These results demonstrate that our methodology offers a sensitive and robust tool to capture facial physiological changes with minimal manual intervention and manual pre-processing of signals. Newer methodological developments for reliable temperature extraction promise to boost IRI use as an ecologically-valid technique in social and affective neuroscience.

## Introduction

Our lexicon is abundant with phrases that ascribe emotions to bodily changes: “pounding heart” for fear, “sweaty palms” for anxiety, or “going red in the face” for embarrassment. These phrases embody various distinct physiological systemic changes. In psycho-physiological research, the measures to capture heart related changes (heart rate-HR) and sweat related responses have been traditionally quantified with well validated measures such as electro-cardiogram (ECG) and skin conductance (galvanic skin response-GSR) respectively. However, the face is a primary region for the expression of emotional states, leading to changes in facial cutaneous blood flow (“blushing” or “turning pale”). In mammals, surface body temperature is constantly influenced by the autonomic nervous system (ANS) through the control of blood perfusion to the surface of the skin, supporting the use of thermal imaging (infra-red-imaging-IRI) in psychophysiological research^1–4^. A thermal imaging technique uses an infra-red camera to capture temperature variations. IRI of face thus has the potential to be a complimentary tool to GSR and HR to quantify physiological status of the body.

Psychophysics measurement techniques are essential to the investigation of the bodily responses that are an integral component of emotional experience^5^ and their dysfunctions in patients with affective disorders^6,7^. However, most conventional psychophysics techniques (GSR, HR) require sensors attached to the body and could compromise the emotional experience and reduce the ecological validity of the experiments^8^. Non-invasive imaging technologies like IRI could overcome the requirement for attaching sensors and improve the ecological-validity of psychophysics studies. Although IRI remains largely unexplored, a few studies have demonstrated its utility in ecologically valid studies to quantify the facial temperature profiles during discrete socio-emotional states^9–11^. However, being contact free, IRI also poses unique methodological challenges, for example motion tracking of the face and the reliable extraction of temperature signals from specific facial regions (nose, cheeks). This has perhaps stalled its widespread adoption in psychophysiological research.

Previous IRI studies of face have mostly relied on manual tracking to locate and extract thermal data, such that investigators manually place regions of interest (ROIs) on the intended region frame by frame^12^. This method depends heavily on clear facial landmarks to guide the manual ROI placement and is thus user dependent and time consuming, especially if data is acquired at a high sampling rate. Other tracking methods have used a cross-correlation template matching to automate tracking but which still requires visual inspection to reliably extract temperature profiles^9,10^. Moreover, these limitations are further exacerbated in ecological experiments if participants are free to move, requiring substantial manual interventions to correct head motion artefacts^9,13^. This has, perhaps, resulted in existing IRI literature focusing mainly on the nose tips, where landmarks are obvious, and is limited and inconsistent for other facial regions like cheeks or forehead where landmarks cannot be easily defined. While recent studies have begun to overcome these limitations using semi-automatic methods, they remain vulnerable to subjective bias and dependent on substantial manual labour.

Recent research in computer vision has made substantial progress in automatic face recognition from images captured in uncontrolled conditions (referred to as “in-the-wild”)^14^. The core step of these techniques is facial landmark detection (detection and localization of certain key points on the face). With recent deep learning algorithms, automatic facial landmark detection can closely match human manual annotation^15–18^. While these approaches are well validated on high-fidelity, full colour facial images, application to thermal imagery has been largely unexplored. Infra-red facial images are sensitive to the surrounding temperature and contrast and they do not provide the clear geometric and appearance patterns of faces that are present in visible spectrum images^19^. This transfer of knowledge from visible spectrum domain to thermal domain is thus a non-trivial problem. Here, we develop a novel deep learning-assisted facial landmark detection method for IRI of the face, which in turn is used to extract thermal signals from the facial regions. To validate our method, we apply it to quantify physiological response induced by a sudden auditory stimulus of a loud sound, which evokes a robust and reliable physiological response^20^. While previous IRI studies have reported temperature decreases in the nose tip^21,22^, comprehensive analyses across facial regions, especially the temporal dynamics, are few and inconsistent^4,22,23^. Hence, we use this new technique here to characterise dynamic changes in temperature across different facial regions (nose-tip, right and left cheeks, forehead) and bench-mark these against conventional GSR and HR measurements.

## Materials and Methods

### Subjects

20 healthy human adult participants (11 females, aged 22–30 years, mean = 25.7 years) were recruited for this study. Written, informed consent was obtained from all participants. All participants had normal or corrected-to-normal vision. Exclusion criteria were habitual cigarette smoking and chronic illness (e.g., cardiovascular or thyroid conditions), psychological disorders (e.g. depression or anxiety) or regular medications assessed by self-report. The study was approved by the Human Research Ethics Committee of QIMR Berghofer and performed in agreement with the Declaration of Helsinki. The subjects were given the choice to withdraw from the study at any time. Each study participant was compensated with an AUD $50 supermarket voucher for their time. Informed consent to publish identifying images (RGB and thermal) was obtained from subject concerned.

### Equipment

The experimental room was kept at a steady temperature and humidity (22 ± 2 °C; 55–70% relative humidity). ECG and GSR recordings were acquired using National Instruments (NI). GSR was recorded with two Ag/AgCL electrodes (0.8–1 cm diameter) filled with a conductive paste and attached to the distal phalanges of the index and ring fingers of the subject’s left hand. GSR was recorded using a standard constant voltage system of 0.5 V and recordings were continuously digitized by an A/D converter with a sampling rate of 2 KHz. To collect ECG data, electrodes were attached to the mid-upper right arm, left wrist, and a mid-upper left arm. To minimize motion artefacts, participants’ hands rested on chair hand-rests. ECG data was also recorded with a sampling rate of 2 KHz.

Infra-red images of face were acquired using a FLIR A615 camera with a 15 mm lens, 640 × 480 pixels, temperature range −20 to 2000 °C and NEDT (noise equivalent differential temperature) < 0.05 °C @ 30°. Emissivity was set at 0.98 and this camera had emissivity correction variable from 0.01 to 1.0. The sampling rate was 5 Hz. Red-Green-Blue (RGB) visible spectrum video images of the face were acquired by Allied Vision PIKE camera, 35 mm lens, and resolution of 800 × 1000 pixels at a sampling rate of 5 Hz.

A novel in-house integrated hardware and software experimentation platform, *LabNeuro*, was used to integrate these multimodal data. CompactDAQ modular IO hardware and software for the system was written using NI LabVIEW and NI Biomedical Toolkit.

### Experimental protocol

**Figure 1:**
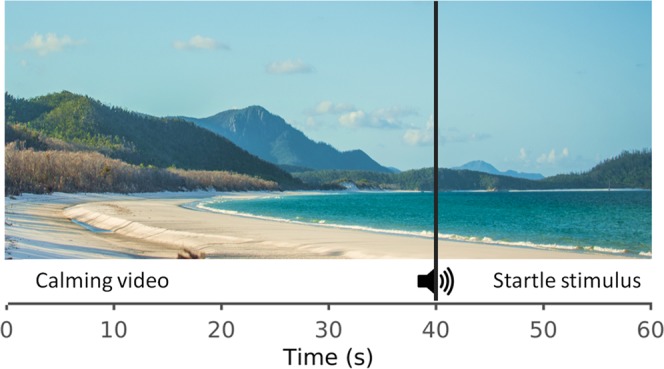
Experimental paradigm. A calming ocean video clip was played for 60 seconds. A loud gunshot sound (80 dB) was played at 40 seconds to mimic a startle response. (An analogous image of the ocean video used for the experiment is shown due to copyright issues).

Subjects were asked to avoid alcoholic and caffeinated beverages for at least 2 hours prior to the experiment to minimize the vasoactive effects of these substances on skin temperature. Testing was only performed in the afternoon between 2–5 pm to avoid potential effects of the circadian rhythm. Prior to the experiment, subjects sat quietly in a chair for about 5 minutes to acclimatize to the experimental setting. Subjects were requested to assume a comfortable posture in the chair while the ECG and GSR electrodes were attached to arms and fingers respectively. The IRI camera and a video camera were then manually focused on the face. The researcher then left the room but retained a visual contact with the participant via a wall-mounted camera. Participants were requested to passively view a 60-second video of ocean waves. A loud gun-shot sound [80 dB (sound pressure level-SPL)] was presented at the 40th second unbeknownst to the subjects (Fig. 1). The calming ocean video was chosen to relax the subject, as there can be spontaneous fluctuations in GSR levels depending on subject’s mental status. Prior reports employing different paradigms, thermal imaging hardware, sampling rate and analytical methodology, varied considerably regarding the latency and recovery of thermal imaging responses^4,21–25^. Twenty seconds post-stimulus was thus deemed to be sufficient time to prompt a thermal response. Stimuli were presented on a 24′ computer screen placed approximately 40 cms in front of the subject. The sound was presented via two loudspeakers each placed beside the stimulus screen. Only one trial of a sudden auditory sound was presented. The duration of the sound stimulus was 1 second with an instantaneous rise (rise time of 0.03 seconds) and fall time of 0.36 seconds.

### Data acquisition and pre-processing

The ECG signal was pre-processed using QRSTool software^26^ to detect the R peaks with the ability to manually correct for missed peaks. Inter-beat interval (IBI) time series was then computed from this and converted to individual subject’s Z-scores. R peak data were further analysed using HRVAS toolbox^27^ to obtain heart rate variability (HRV) frequency domain measures. These were calculated via the auto-regressive method using a window size of 16 seconds, with 15 samples overlap, nfft of 1024 and cubic spline interpolation rate of 2 Hz. Time-frequency decompositions of IBI are typically categorised as high frequency (HF) from 0.15 to 0.4 Hz, low frequency (LF) from 0.04 to 0.15 Hz, and very low frequency (vLF) from 0.003 to 0.04 Hz^28^. HR data metrics were computed for the whole 60 seconds but analyses focussed upon a 5 second baseline interval before the audio stimulus and the 10 second window immediately post-stimulus.

GSR data were extracted and pre-processed offline using custom programs in MATLAB (The Mathworks, USA). The GSR signal was de-trended and low pass filtered at 5 Hz using a zero phase FIR filter EEGLAB^29^ to remove motion artefacts. To minimise inter-subject variability in skin conductance, GSR signals were converted to individual Z-scores.

The facial thermal images were visually inspected to assess quality. The image of each thermal video frame was converted to a .MAT file of 640 × 480 pixels with each pixel having a temperature value precise to 2 decimal places.

### Thermal image data extraction and analysis

**Figure 2:**
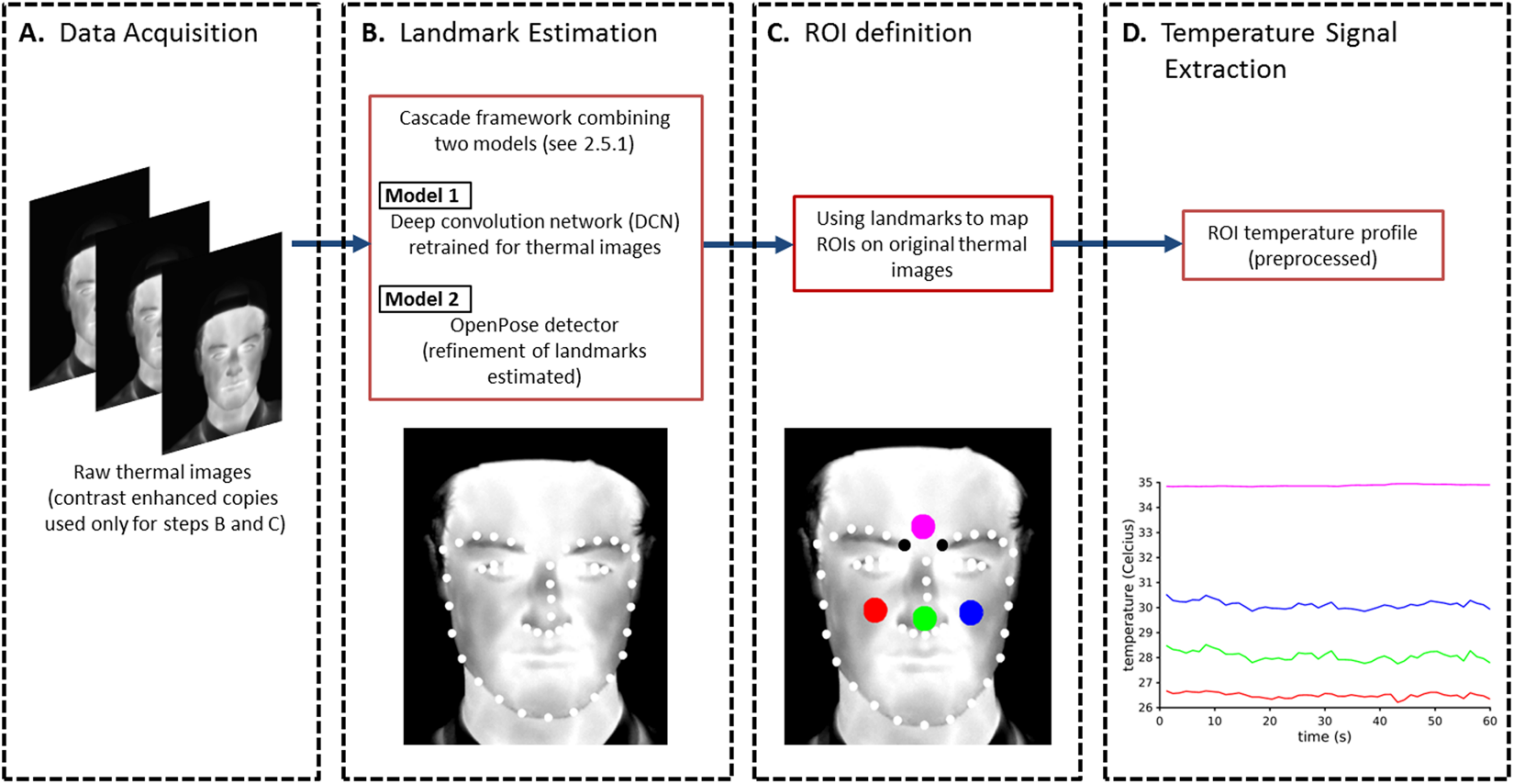
The framework for the landmark-based methodology to extract temperature profiles from regions of interest. (**A**) Thermal video of facial responses is acquired. (**B**) Facial landmarks are located automatically on the contrast-enhanced thermal images. For this, model 1 and model 2 are used in cascade framework (**C)** Four regions of interest (ROI) are defined relative to two medial eyebrow landmarks (black dots). (**D)** Temperature profiles are extracted from each ROI based on the mean of the temperature values inside the ROI and pre-processed.

A block diagram of the method is displayed in Fig. 2. In each sampled frame of the thermal video, key anatomical points on the face, called *landmarks*, were detected using a framework that combines two artificial neural networks (Section 2.5.1). The midpoint between the two medial eyebrow landmarks was used as the reference to define four ROIs for each subject (Section 2.5.2). Finally the mean temperature in each ROI was computed at each frame, and these four temperature time series, after pre-processing, were used to statistically compare pre- and post- stimuli data. RGB images acquired, at the same time as the thermal images, were not used for thermal signal extraction and were only used for comparison of landmark detection performance on thermal images.

#### Facial landmark estimation

Facial landmark estimation techniques are a family of computer vision methods which automatically locate key anatomical points of the face, outputting *X* and *Y* coordinates of these landmarks in each video frame. To apply these techniques, a copy of each thermal image was first enhanced by normalising its range to the interval [0,1] and increasing the contrast using the MATLAB (The Mathworks, USA) imadjust() function with gamma = 1.0. Pilot testing showed superior landmark detection using this adjustment. The original copies of raw images were retained for temperature measurement.

**Figure 3:**
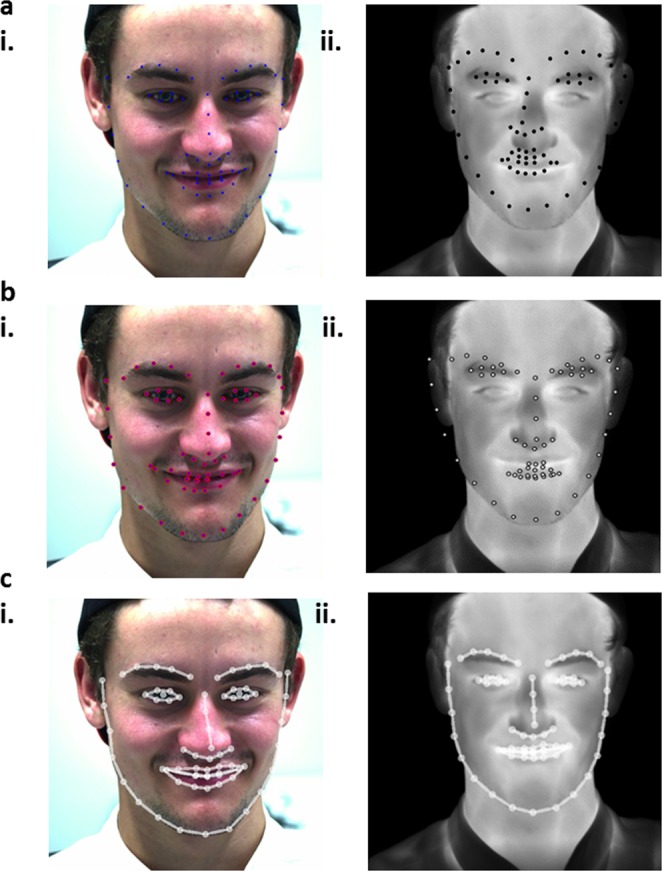
Facial landmark estimation. Selected samples of the landmarks estimated for (i) RGB and (ii) thermal images recorded synchronously. (**a**,**b)** Points detected using existing RGB facial landmark estimators illustrate the inaccuracy when RGB-trained systems are applied to thermal data (**a)** Dlib C++ Library^31,32^. (**b)** OpenFace^30^). (**c)** The cascaded-framework presented in this paper; for RGB and thermal images.

We first applied existing benchmark methods based on deep convolutional neural networks (DCNNs) trained for facial landmark localization using RGB (visible spectrum – red, green, and blue light) training images^30–32^. However, these benchmark methods failed to detect landmarks correctly when applied to the IR images (Fig. 3a,b).

Previous work on infrared based facial analysis and ROI tracking primarily explored the use of standard machine learning techniques^33–36^. These models allow optimal landmark detection in some cases but need further improvement as they rely on data attributes (features) which in the case of IR facial images lack the details present in visible spectrum images. Therefore, it is necessary to combine features from visible images and thermal images for facial analysis. Hence, we employed this in our second approach and incorporated two landmark estimation models in a cascade framework which proved successful.

Model 1: We implemented a deep learning landmark estimation system first trained on publicly available RGB images and performed further feature learning to fine-tune it (*i*.*e*. to automatically adapt the learned parameters) for thermal images. Specifically, a framework known as task-constrained deep convolutional network (TCDCN) framework was used first, which is a facial landmark estimator system that returns precise landmark estimations of the face. It uses head pose estimation or facial attribute inference as supplementary information for robust landmark estimation. The TCDCN was pre-trained with images annotated with five landmarks then fine-tuned to predict the dense landmarks of 68 facial points. The feature extraction stage contains 4 convolutional layers, 3 pooling layers, and 1 fully connected layer. The TCDCN model was trained and tested with the RGB image databases as mentioned in a previous study^37^. Subsequently, we performed a fine-tuning of the earlier learned filters by further training the network with labelled thermal images. For this purpose, we used public thermal image databases^33,38^ as labelled training data. We first applied a face detector to the images to provide an initial configuration of the landmarks. The anatomical points labelled for landmarking on the thermal training data sets were consistent with the points used in a number of current RGB face image databases, allowing efficient re-training of the existing model.

Model 2: We used the OpenPose detector^39^, which locates facial landmarks by a recently developed method^40^ that employs a robust multi-view bootstrapping architecture. This model outputs a confidence map (an image where the value at each pixel indicates the certainty of the detector) for each facial landmark and the final estimated position for each landmark is obtained by finding the maximum of the confidence map.

These two models were then combined to improve the accuracy of the facial landmark detection and tracking across videos. This cascade framework follows previously established approach^37,41^. First, we use model 1 to detect the landmarks. Then, we eliminate all detected points for which the detection confidence score is lower than 0.65. After that, we perform a landmark detection refinement using the model 2. Where valid detections are recorded using both methods, the refined point locations are the average of the points detected by the two models. If neither detector is able to locate a point with sufficient confidence, then we use the landmark locations with the highest score among both models. The output for each frame is a set of 70 facial landmark locations, derived from the confidence maps for each detected keypoint. These 70 landmarks correspond to the Multi-PIE 68 point mark-up^42^, with the other two points being the centres of the pupils.

To improve the stability of the facial landmark detection across frames, we performed a final point correction similar to the method previously employed^30^. This uses multiple initialization hypotheses at different orientations and picks the final location with the maximum likelihood. If the estimated location in the current frame was within five pixels of the previous location, the point was labelled as valid. If a point was rejected, the nearest suitable point inside the five pixel radius was selected.

#### Region of interest (ROI) location

The medial eyebrow landmarks showed the most consistent and reliable detection confidence values among all the landmarks and were hence chosen as anchor points for ROI definition. The midpoint between the two medial eyebrow landmarks was used as the reference point to define four ROIs: forehead, right cheek, left cheek and nose-tip. The size of all ROIs was a circle of 10-pixel radius. Figure 2C illustrates the location of the ROIs.

#### Extraction of temperature profiles

The mean temperature across all pixels in the ROI was calculated per frame. For the frames in which landmarks were not detected, temperature values from previous frame’s ROIs were used. Each time series was then corrected for outliers (any point more than 5 standard deviations from the mean of the time series), replacing these using cubic spline interpolation. The resulting signal values were converted to the individual subject’s Z-scores. For statistical comparison, the pre-stimulus duration of 5 seconds and post-stimulus interval of 10 seconds was used. A post-stimulus duration of 10 seconds for comparison is justified as previous investigation on facial temperature response to startle response indicated a latency as quick as 300 milliseconds^4^ while other reports suggested a response of around 10 seconds^3^. Significance testing for changes in HR responses, GSR and thermal responses was performed using paired t-tests on the mean values for pre- and post- stimulus.

## Results

Landmarks could not be reliably and accurately estimated for 3 subjects and landmark estimation was lost at critical periods post-stimulus for 2 other subjects. Hence data from 15 participants were used.

### Facial landmark accuracy

The accuracy of the facial landmark estimation was assessed against a ground truth by comparing its performance on a randomly selected input frames to manually annotated landmarks using a previously validated method^37^. We randomly selected the IR facial images of one of the subjects on which to perform this analysis. A landmark point was considered correctly detected if the distance between the predicted and the ground truth location was within five pixels. The cascade-framework reached an average accuracy of 92%, *i*.*e*. 92% of all facial points were correctly detected to within 5 pixels of the ground truth on the test images. The accuracy was even higher if points from eyes, nose and outer-mouth were excluded (96% for the 33 points). Figure 3c demonstrates the facial landmark detection using various algorithms.

### Physiological changes

#### Increases in GSR and IBI

**Figure 4:**
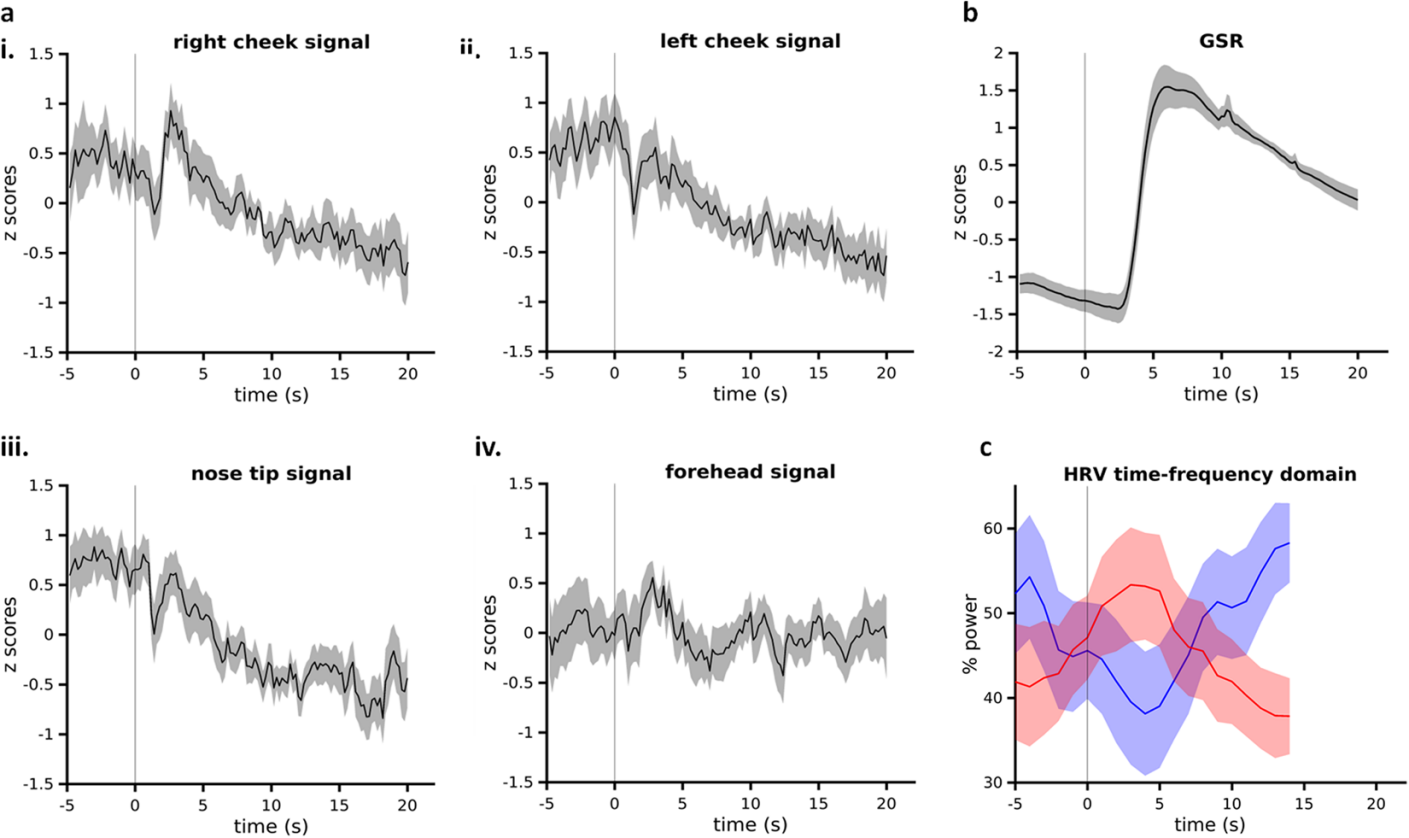
Physiological responses to the sudden auditory stimulus. (**a)** Temperature signals extracted from the right (i) and left cheek (ii), the nose-tip (iii) and forehead (iv). (**b)** GSR. Time is indicated on x-axis and z-scores indicated on y-axis. Time is indicated on x-axis and z-scores indicated on y-axis **(c)** HRV: high frequency component (HF HRV) (blue), low frequency component (LF HRV) (red). Time indicated on x-axis (note different time scaling: data not computed for the last 7 seconds to avoid edge effects in frequency decomposition) and percentage power on y-axis. Vertical line at 0 seconds indicates the onset of auditory sound stimulus. Shading indicates SEM.

**Figure 5:**
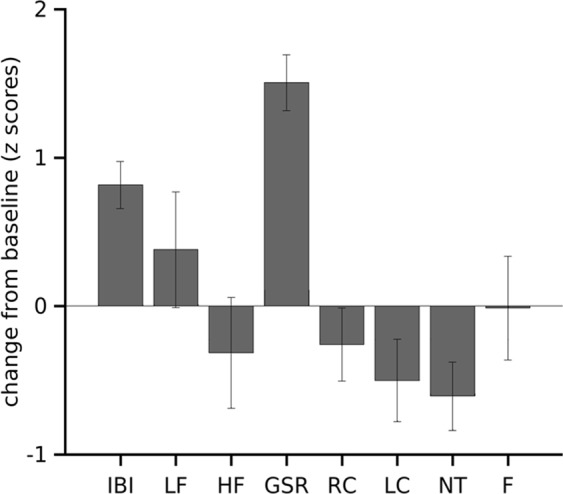
Change in physiological signals induced by auditory stimuli. Mean change of 10 seconds post-stimulus from baseline (5 seconds pre-stimulus stimulus) for all physiological measures used in this study. IBI: inter-beat interval, LF HRV: low frequency heart rate variability, HF HRV: high frequency heart rate variability, GSR: galvanic skin response, RC: right cheek temperature signal, LC: left cheek temperature signal, NT: nose tip temperature signal, F: forehead temperature signal. Error bars indicate SEM.

Skin conductance showed a robust increase post-stimulus across all the subjects. This increase peaked between 5–10 seconds post-stimulus, and was significant compared to the 5 seconds pre-stimulus interval (Figs 4b, 5; Table 1). Skin conductance gradually decreased back to baseline after 10 seconds.

**Table 1:** Statistical analysis to compare baseline and post-stimulus condition. IBI: inter-beat interval, LF HRV: low frequency heart rate variability, HF HRV: high frequency hear rate variability, GSR: galvanic skin response, RC: right cheek temperature signal, LC: left cheek temperature signal, NT: nose tip temperature signal, F: forehead temperature signal.

Modality	Mean		SEM		T stat	p value	Effect size
	Pre-stimulus	Post-stimulus	Pre-stimulus	Post-stimulus			
IBI	−0.40	0.36	0.18	0.15	−5.14	0.0001***	1.22
LF-HRV	0.15	0.53	0.25	0.19	−0.97	0.32	0.43
HF-HRV	−0.22	−0.54	0.23	0.19	0.84	0.34	0.38
GSR	−1.19	0.31	0.13	0.18	−7.99	0.00001***	1.55
RC	0.43	0.17	0.21	0.11	1.29	0.31	0.39
LC	0.62	0.12	0.23	0.11	2.18	0.09	0.80
NT	0.72	0.11	0.20	0.11	2.63	0.02*	0.89
F	0.04	0.03	0.28	0.12	0.03	0.97	−0.01

***p < 0.001, **p < 0.01, *p < 0.05. Thermal data corrected for multiple comparisons (FDR, *p* = 0.05).

The IBI showed a significant increase 10 seconds post-stimulus as compared to 5 seconds pre-stimulus (Fig. 5; Table 1). In addition, frequency domain HRV also showed changes, with LF HRV decreasing and HF HRV increasing following the auditory stimulus, although these changes were not statistically significant (Figs 4c, 5; Table 1). This trend reversed after 10 seconds.

#### IRI response to auditory stimulus

We then examined the IRI responses to the auditory stimulus. Visual inspection of the time series for all the ROIs showed an irregular increase/decrease in temperature immediately post-stimulus. Subsequently, a gradual decrease in temperature was observed. Statistical comparison of the average signals in the 10 second post-stimulus window was significantly lower than in the 5 seconds pre-stimulus baseline in the nose tip (Figs 4a, 5; Table 1). The signal of the left cheek, right cheek showed a similar trend but did not reach significance whereas forehead signal did not show a substantial change (Figs 4a, 5; Table 1).

#### Latency of IRI responses compared to GSR

**Figure 6:**
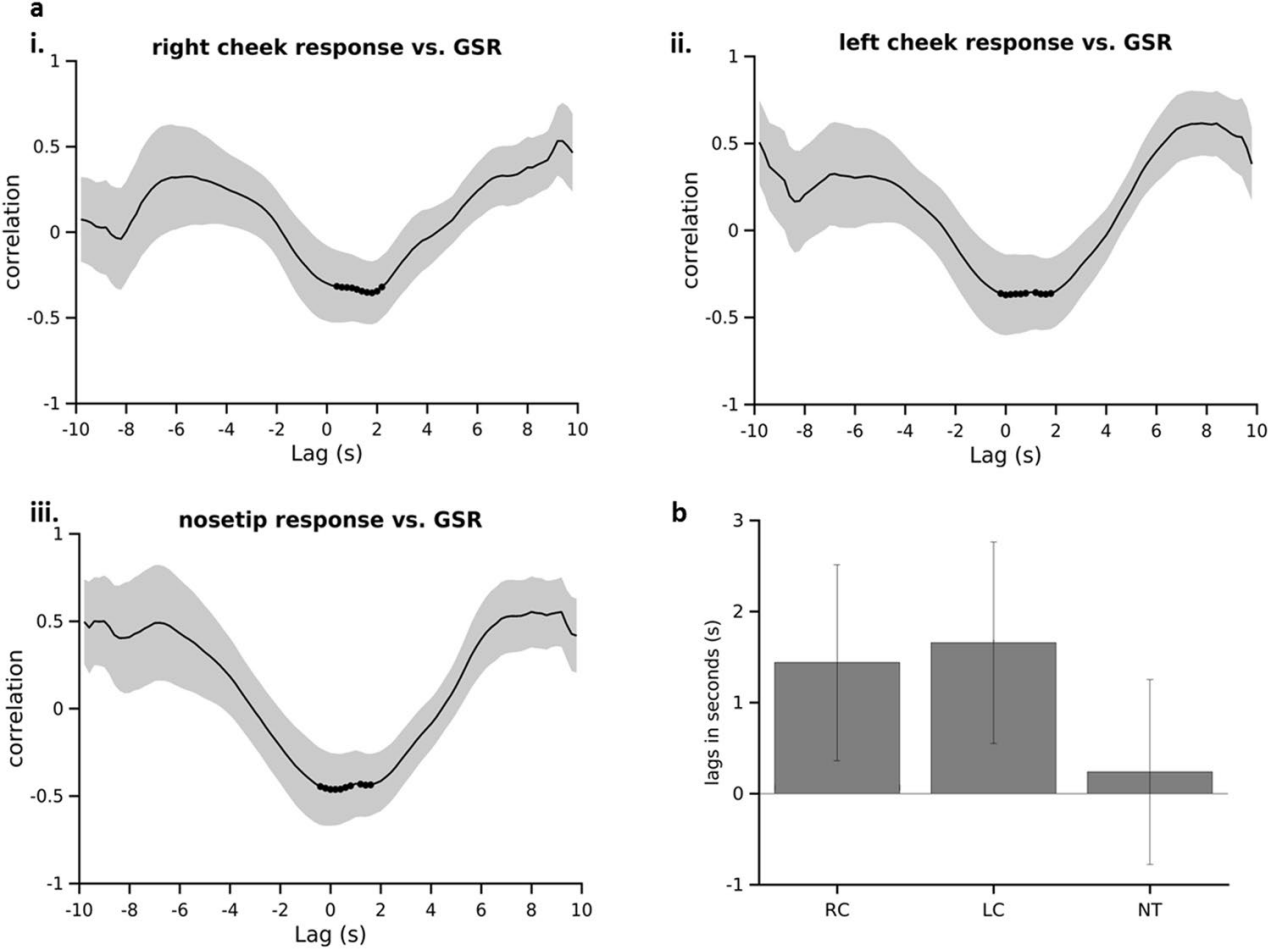
Cross-correlation of thermal responses versus (vs.) GSR (**a**) Cross correlation between IRI and GSR responses for 3 ROIs for 10 seconds post-stimulus. (i) right cheek, (ii) left cheek, (iii) nose tip. Black dots on the plots represent 10 minimum values of correlation. Shading indicates SEM (**b**) mean of time lags between temperature response and GSR corresponding to minimum 10 values of cross-correlation. RC: right cheek temperature signal, LC: left cheek temperature signal, NT: nose tip temperature signal. Error bars indicate SEM.

Previous studies suggested that facial thermal responses were delayed^43^. However, using our algorithm, we sought to further quantify the relative temporal dynamics of facial thermal responses. We applied cross-correlation analysis to the facial thermal response and GSR response curves (10 seconds post-stimulus) in our dataset, to determine the relative time delay (phase lag) between the onsets of auditory stimulus induced changes in two signals. The time lag between the response onsets in signals is indicated by the time point at which the cross-correlation value is maximum for positively correlated signals or minimum for negatively correlated signals. Nose-tip and bilateral cheek responses were used for this analysis as the forehead thermal response showed minimal change in temperature post-stimulus. The average correlation curve showed minimum correlation at time lags of around 0–2 seconds (Fig. 6a). This lag was minimal for nose-tip (Fig. 6b). These results demonstrated that thermal response onset latency are only slight delayed compared to the GSR (<2 s).

## Discussion

In this study, we developed a novel method to extract the dynamic temperature profiles from key facial regions using deep learning and applied this to investigate the physiological responses to a sudden auditory stimulus. We found 1) our face tracking and temperature extraction methodology worked reliably without motion correction of the facial images but rather adaptive landmark detection; (2) minimal manual requirement for initial placement of ROIs; (3) a continuing, regionally specific decrease in temperature for up to 15 seconds in response to auditory stimulus; (4) a short delay of approximately 2 seconds of thermal responses in comparison to GSR.

### IRI response

Blood flow changes are visible as specific facial colour patterns perhaps assisting in decoding of emotion^44^. For instance, fear causes paling of face, anger can cause flushing of facial skin^45,46^ and embarrassment is known to cause blushing^47,48^. These perfusion changes are caused by blood re-distribution via cutaneous blood vessels which are richly innervated by sympathetic nerves. The sympathetic activity thus seems to be responsible for the rich repertoire of subtle physiological changes induced by emotions.

Many previous studies have used an acoustic stimulus as part of the acoustic startle paradigm^4,23,49,50^. Responses induced by these stimuli could be considered a case of “fight-or-flight” reaction in response to environmental stressors mediated by the sympathetic nervous system^49,51^. As part of this response, sympathetic nerve fibres trigger an increase in heart rate and blood pressure^52^, as an animal’s defence mechanism for survival and thus heightening blood flow to the musculoskeletal system and other essential tissues. In this study, we induced a response with a sudden acoustic stimulus. Although we did not record EMG activity to know with certainty if the response induced was indeed a startle response, a sudden loud sound was used as stimulus to mimic such a physiological reaction.

Physiological changes mentioned above are consistent with peripheral blood blow being directed towards the major organs of the body. Previous studies have reported a decrease in nose, maxillary and cheek temperature and increases in peri-orbital and supraorbital temperature^2,22^. Our results are, thus, consistent with the temperature decreases in nose-tip, left cheek and right cheek regions in response to such a sudden stimulus.

GSR signals are considered the gold standard in peripheral neurophysiological and psycho-physiological studies, providing a valuable benchmark for IRI research. GSR has a rapid response profile with a delay of 1–3 seconds after stimulus onset^53^. In earlier studies, thermal responses have been seen to be sluggish. For instance, thermal response latency in non-human primates have been reported to be around 10 seconds^3^. However, that study used data points at 10 second time windows, compromising the temporal accuracy of the response profiles. Merla and colleagues (2007) reported a facial thermal latency of approximately 4–6 seconds compared to GSR latency^24^. Pavlidis *et al*. (2001) studied the thermal response of peri-orbital regions underlying the startle response and suggested that temperature changes were observed within 300 milliseconds^4^ but they presented data from only one point in time before and one point after stimulus presentation and reported no statistical significance of temperature differences. Overall, these diverse findings – with differences in stimuli and analysis methods – make it difficult to reach a definite conclusion about the facial thermal response latency compared with GSR. With the improved algorithm for facial temperature extraction employed in this study, the detailed temporal profiles of facial thermal responses were comparable to that of GSR. Nose-tip thermal response latency was similar to that of GSR (around 2–3 seconds after stimulus) and the cheek thermal latency was about 1–2 seconds lagging behind that of GSR.

Nose-tip showed the most robust and rapid thermal response when compared to cheeks and forehead. Previous studies have also reported nose-tip to show consistent thermal variations in response to emotional activations^2^. Anatomically, nose-tip is unique among facial regions being devoid of subcutaneous facial muscles. It is thus less affected by artefacts due to muscle contraction than the cheeks and forehead. Moreover, it is well vascularised with abundant arteriovenous anastomoses making it sensitive to subtle blood flow variations^54,55^. On the other hand, airflow during breathing may cause temperature changes at nose-tip. Recording of ventilation or breathing rate should additionally be undertaken in future studies to address this. Therefore, examining thermal responses across different facial regions is thus recommended to confirm if the temperature changes induced by the stimuli are consistent.

### HR response

Resting HR is slower than the intrinsic pace-making activity, reflecting predominant inhibitory control from the parasympathetic system and thus a dominant high frequency component (Fig. 4c). In response to the sudden auditory stimulus, we found an immediate heart rate decrease and that the balance of HF and LF components reversed for up to 10 seconds. This is in line with previous findings that initial stages (within seconds) of startle response are characterized by bradycardia^56–58^. Subsequently as threat becomes more imminent, the heart rate decrease reverses the direction of change to cardiac acceleration by either sympathetic increase or parasympathetic withdrawal^59^. Our frequency domain results show increase in LF component suggesting former whilst simultaneous decrease in HF component suggesting the latter. These components reversed to baseline level after 10 seconds.

### Novel face tracking and temperature extraction methodology

The primary advantage of thermal imaging in psycho-physiological research is its contact free property. Free movements, however, make data extraction and signal processing challenging. Here, we addressed this issue using landmark detection algorithm trained on publicly available RGB and thermal images for use on thermal images acquired in our study. Using these landmarks for ROI placement for thermal signal extraction required minimal manual intervention which was specifically needed only once on the first frame for each subject. This method also enabled temperature extraction from multiple regions of the face. Overall, our work established a semi-automated pipeline for thermal imaging analysis, requiring much less manually intervention than previous studies^1,9,10,13^.

Furthermore, the temperature signals were minimally pre-processed without the need of excessive filtering or smoothing, demonstrating the robustness of our methodology to capture meaningful physiological changes. Substantial smoothing has often been employed in previous studies^9,13^, to improve signal to noise ratio and remove breathing related artefacts. Breathing can affect the temperature signal of the face, especially the nose-tip. Previous efforts to overcome this problem involve low-pass filtering the signal below 0.15 Hz to avoid breathing-related oscillations^13^. We chose not to use such aggressive filtering techniques as it likely removes relevant signals. Interaction between startle reflex and respiration has been shown^50^ and thus its effects on blood perfusion of face, could still be a part of the relevant signal following the startle stimulus. However, a previous study showed that even when respiration rate changed two-fold as a result of heavy breathing, no temperature change was observed on the nose of monkeys^43^. None the less, in the absence of strong evidence, a cautious approach argues against strict low-pass filtering of thermal imaging data to remove breathing artefacts.

## Limitations

We recorded the thermal and physiological measures for 20 seconds post-stimulus. While this allows quantification of response onset in this study, longer post-stimulus recording would have been beneficial for characterizing the recovery of thermal responses.

The thermal signals from all the regions showed a spike-like increase or decrease 1–3 seconds post-stimulus. Although we are uncertain about the cause of this, use of a high sampling rate of 10 Hz or above may be able to determine if this is in fact an immediate physiological response. Alternatively, this could be attributable to motion artefacts induced by the sudden auditory stimulus but which requires further investigation.

We have shown that facial landmarks are consistently detected across the thermal images in the majority of participants. However, landmark detection failed in some subjects, perhaps because their temperature profiles were quite different from the training data. This suggests that a larger training dataset with a variety of thermal patterns may significantly improve performance. With the introduction of a new public database of annotated high resolution thermal face images^60^, the robustness afforded by our system could be further enhanced. Additionally, other machine learning techniques such as deep transfer feature learning^61^ may help to improve performance. Transfer feature learning approaches aim to adapt models from one domain to another (*i*.*e*. visible images to thermal) with minimal data for the new domain. This may reduce the need for large thermal image training data sets by transferring a model trained on a very large visual domain corpus to the thermal domain more effectively than the fine tuning approach used in this work.

## Conclusion and Future Directions

IRI is an exciting new technique in the tool kit of a psycho-physiologist. Use of thermal imaging of the face offers new avenues for face-specific physiological changes, such as flushing with anger of blushing in embarrassment. Its contact-free nature and hence advanced ecological validity encourages for its wide use in affective research. Reliable, easy and efficient extraction of facial temperature still limits its widespread use. However, as we have demonstrated, using information from visible spectrum images is an efficient way to help extract reliable and sensitive facial temperature profile from thermal images using advanced machine learning algorithms. Furthermore while existing studies are predominantly focused on specific ROIs, physiological responses in other regions of the face could also be informative. Future work could look into spatial decomposition of thermal signals from all facial regions for a compressive investigation on facial thermal physiology.
